# Climate-change-induced range shifts of three allergenic ragweeds (*Ambrosia* L.) in Europe and their potential impact on human health

**DOI:** 10.7717/peerj.3104

**Published:** 2017-03-16

**Authors:** Karen Rasmussen, Jakob Thyrring, Robert Muscarella, Finn Borchsenius

**Affiliations:** 1 Section for Ecoinformatics & Biodiversity, Department of Bioscience, Aarhus University, Aarhus C, Denmark; 2 Astma-Allergi Danmark, Roskilde, Denmark; 3 Arctic Research Centre, Department of Bioscience, Aarhus University, Aarhus C, Denmark; 4 Science Museums, Aarhus University, Aarhus C, Denmark

**Keywords:** Aeroallergen, Habitat suitability, MAXENT, Public health, Urban heat islands, Allergy, *A. artemisiifolia*, Species distribution models, Invasive species, Climate change

## Abstract

Invasive allergenic plant species may have severe health-related impacts. In this study we aim to predict the effects of climate change on the distribution of three allergenic ragweed species (*Ambrosia* spp.) in Europe and discuss the potential associated health impact. We built species distribution models based on presence-only data for three ragweed species, using MAXENT software. Future climatic habitat suitability was modeled under two IPCC climate change scenarios (RCP 6.0 and RCP 8.5). We quantify the extent of the increase in ‘high allergy risk’ (HAR) areas, i.e., parts of Europe with climatic conditions corresponding to the highest quartile (25%) of present day habitat suitability for each of the three species. We estimate that by year 2100, the distribution range of all three ragweed species increases towards Northern and Eastern Europe under all climate scenarios. HAR areas will expand in Europe by 27–100%, depending on species and climate scenario. Novel HAR areas will occur mostly in Denmark, France, Germany, Russia and the Baltic countries, and overlap with densely populated cities such as Paris and St. Petersburg. We conclude that areas in Europe affected by severe ragweed associated allergy problems are likely to increase substantially by year 2100, affecting millions of people. To avoid this, management strategies must be developed that restrict ragweed dispersal and establishment of new populations. Precautionary efforts should limit the spread of ragweed seeds and reduce existing populations. Only by applying cross-countries management plans can managers mitigate future health risks and economical consequences of a ragweed expansion in Europe.

## Introduction

Climate is one of the most important determinants of species distributions at regional and global scales (Parmesan & Yohe, 2003; Burrows et al., 2011). Human-induced climate change is affecting global vegetation patterns, ecosystem functions and the distribution range of multiple terrestrial and aquatic species (Cramer et al., 2001; Gonzalez et al., 2010). One dramatic effect of climate change is the shift in species distribution range and phenological interactions (Parmesan & Yohe, 2003; Visser & Both, 2005), which can change the pollen season and lead to increases in local pollen production (Rogers et al., 2006). Allergic responses such as rhinitis (hayfever) and asthma, caused by plant pollen are therefore globally an increasing public health concern (Albertine et al., 2014; Essl et al., 2015; Hamaoui-Laguel et al., 2015; Schindler et al., 2015).

Among allergenic plants, ragweeds (*Ambrosia* L., Asteraceae) are considered to be among the most potent aeroallergens. *Ambrosia* is distributed from Mexico to Canada and contains at least 40 species (Essl et al., 2015). Ragweed species flower from July to October and each plant is able to produce millions of wind-dispersed pollen grains that may be transported over long distances, with reports ranging to over 1,000 km (Belmonte et al., 2000; Bullock et al., 2013; Hamaoui-Laguel et al., 2015). Ragweed pollen is a significant cause of allergic reactions (Frenz, 2001; Ziska et al., 2011). The threshold for provoking allergic rhinitis in ragweed pollen sensitized patients is below 20 grains m^−3^, and in sensitive patients be as low as 1–5 grains m^−3^ (Déchamp et al., 1997; Šikoparija et al., 2009) compared to 50 grass pollen grains (Davies & Smith, 1973). Ragweed pollen furthermore induces asthma about twice as often as other types of pollen (Skjøth et al., 2010). In this regard, common ragweed (*A. artemisiifolia*), perennial ragweed (*A. psilostachya*) and giant ragweed (*A. trifida*) are of special concern as they rank among the most widespread allergenic plants (Bassett & Crompton, 1982; Ghosh et al., 1994; Rich, 1994; Frenz, 2001). The medical cost of allergies in the United States is estimated to an annual cost in excess of $18 billion a year (Centers for Disease Control and Prevention, 2011). Ragweed is the major cause of late summer allergic rhinitis (hay fever) in the United States and Canada (Arbes Jr et al., 2005), and is also a significant problem in Southern and Southeastern Europe where allergy is the most common chronic disease (Papadopoulos et al., 2012). Estimated medical costs of ragweed-related allergies are substantial in the affected countries. In Hungary, where 25% of the population is allergic to ragweed, Kazinczi et al. (2008) estimated the annual cost to exceed €110 million per year; in Austria Gerber et al. (2011) put the cost around €88 million per year (for a comprehensive review, see Task 5 in Bullock et al. (2013)).

Ragweed populations remain relatively rare in Northern Europe, where low autumn temperatures prevent ragweed species from flowering and reaching reproductive maturity (Chapman et al., 2014). However, global warming is predicted to facilitate a northwards expansion of ragweed species (Storkey et al., 2014; Essl et al., 2015). This can accelerate the establishment and pollen production of self-propagating ragweed populations, and extend the local pollen season in regions currently climatically unsuitable (Gerber et al., 2011; Cunze, Leiblein & Tackenberg, 2013). Increasing amounts of anthropogenically released carbon dioxide (CO_2_) also has the potential to increase pollen production (Sheffield, Weinberger & Kinney, 2011). Studies have shown an increase of up to 61% in ragweed pollen production in a CO_2_ enriched atmosphere, both in climate-controlled greenhouses and in field based experiments (Ziska et al., 2003; Rogers et al., 2006). Furthermore, Patz & Olson (2006) described how especially plants in urban areas are exposed to elevated CO_2_ concentrations, i.e., from industry and dense traffic. Cities are therefore prone to a disproportionate increase in ragweed pollen production, with serious consequences for public health (Ziska et al., 2003). This raises concern about the consequences of the increasing frequency of which ragweed species have been registered across continental Europe, where they are now considered bioinvaders (Fernández-Llamazares et al., 2012). Over future decades, ragweed may become a major source of pollen allergy in Northern Europe, with severe medical and economic implications (Bullock et al., 2013).

In this paper, we use species distribution modeling (SDM) to explore which areas of Europe are most susceptible to successful invasion of common ragweed, perennial ragweed and giant ragweed by year 2100 under two different IPCC climate scenarios (RCP 6.0 and RCP 8.5). We show that all three ragweed species are expected to expand and shift their potential distribution across Europe by year 2100, and that ragweed species by the end of the century may cause large-scale health related impacts on the European continent.

## Methods

### Study species and occurrence records

We focused our analyses on the three most widespread ragweed species in Europe: common (*Ambrosia artemisiifolia*), perennial (*A. psilostachya*) and giant (*A. trifida*) ragweed. Common ragweed is widespread in large parts of Eurasia and North America and is considered highly invasive across the globe (GISD, 2012). Outside North America, perennial ragweed is found in counties across Europe and in Australia, and giant ragweed is widely recorded in Europe, Asia and South America. A map and table displaying the distribution of the three species in Europe is provided in Fig. S1 and Table S1. Ragweed species have been known in Europe since the 19th century and are now naturalized in more than 20 European countries (Gerber et al., 2011; GISD, 2012; Essl et al., 2015).

All three studied ragweed species are native to North America. Geo-referenced occurrence records from North America (Table 1) were therefore downloaded for each species from the Global Biodiversity Information Facility online database (GBIF; http://www.gbif.org). All records with obviously erroneous coordinates (e.g., records located in sea) were excluded. To ensure that their northern distribution limits were well represented in the data, all non-georeferenced records from Canada that had sufficiently precise locality information were geo-referenced using Google Earth and added to the dataset. To verify the precision of the occurrence data, the elevation of each record was extracted in ArcGIS 10.1 (ESRI, Redlands, CA, USA) using a topographical map at 10 arc-minutes resolution obtained from the WorldClim database version 1.4 (http://worldclim.org; Hijmans et al., 2005). If the difference between the extracted elevation and that cited in the original record exceeded 100 m then the occurrence point was removed from the dataset to avoid inaccurate assessment of climate variables at the collection site due to imprecise geo-referencing (Skov & Borchsenius, 1997).

**Table 1:** Distribution records used for modeling of common ragweed (*Ambrosia artemisiifolia*), perennial ragweed (*A. psilostachya*) and giant ragweed (*A. trifida*).

	Common ragweed	Perennial ragweed	Giant ragweed
North American occurrences	2,284	1,531	1,985
European occurrences	4,127	248	364
Total	6,411	1,779	2,349

**Notes.**

The dataset include North America distribution records from the GBIF online database (http://data.gbif.org). MAXENT automatically removed duplicated presence records defined as records in the same 10’ grid cell.

### Bioclimatic variables

Three of the most important climatic determinants of global vegetation patterns are minimum temperature, growing season and water balance (Prentice et al., 1992; Normand, Svenning & Skov, 2007). We therefore selected these three variables to model occurrence patterns of ragweed over the next century: growing degree days (GDD), absolute minimum temperature of the coldest month (*T*_min_) and water balance (WBAL) (for further information on the climatic variables see Appendix S1). We used two IPCC AR5 climate change scenarios, including a high-end (RCP 8.5) and a moderate (RCP 6.0) climate change scenario, to represent future climate conditions by year 2100 (projection period 2070–2099).

Because MAXENT implicitly assumes that grid cells have the same size when choosing random samples, equal area projections are preferable (Elith et al., 2011). Therefore, prior to analyses, we reprojected the climatic layers onto a Behrmann’s equal area projection, using a bilinear interpolation, with datum WGS1984 and a grid size resampled to 10 km^2^. We used ArcGIS 10.1 for resampling and reprojection of the environmental layers.

### Species distribution modeling

The potential future distribution models were based on the occurrence records from North America. This approach is considered the most conservative estimate for the habitat suitability, and therefore is generally applied to model the distribution of invasive species (Guisan & Thuiller, 2005; Pearson, 2007). Ragweed species potentially undergo niche shifts after introduction to Europe. In this case, distribution models based on the native range only (i.e., North America) may underestimate the potential distribution in Europe. Therefore, we also built models based only on occurrence records from the invasive range in Europe and on combined records from North America and Europe, and we present results from those models in Table S2 and Figs. S2 and S3. Preliminarily results based on AUC values indicated that all models performed as adequate modeling tools (AUC > 0.7) (Table 2 and Table S2). However, when comparing the results of the models we found models trained on the native range made the best prediction of where the *Ambrosia* species currently occur abundantly in Europe (Essl et al., 2015). Models based on combined invasive and native range or invasive range only data tended to overpredict habitat suitability towards the north of Europe, e.g., areas such as Southern Scandinavia, where flowering and seeding of these species is currently almost absent (Figs. S2 and S3). Therefore, we base our main findings on models built with occurrence records from native range only (i.e., North America).

**Table 2:** Model predictive ability under current climate based on median Area Under the Curve (AUC) values for common ragweed (*Ambrosia artemisiifolia*), perennial ragweed (*A. psilostachya*) and giant ragweed (*A. trifida*). AUC values were derived from average test AUC values for MAXENT models of 15 replicates based on all occurrence records from the native range (see Table 1).

	Model AUC (based on test records in native range)		
	Common ragweed	Perennial ragweed	Giant ragweed
Median ± SD	0.797 ± 0.013	0.806 ± 0.014	0.817 ± 0.012
Min–Max	(0.774–0.825)	(0.787–0.827)	(0.797–0.837)

After building models based on current climate conditions, we projected the models into the projected climatic conditions for Europe (defined as areas 34–72°N and 11°W–32°E) to predict future potential ranges of the three species in that continent. Models for all three ragweed species were modeled using MAXENT v3.3.3k (http://www.cs.princeton.edu/ schapire/maxent), a machine-learning method that uses the principle of maximum entropy to approximate the unknown probability distribution of a species based on presence-only data (Phillips, Anderson & Schapire, 2006). The output of MAXENT consists of values proportional to the expected number of species occurrences in each grid cell (relative occurrence rate or ‘habitat suitability’). We chose to use MAXENT because it has been shown to outperform other presence-only methods (Elith et al., 2006; Phillips & Dudik, 2008; Elith et al., 2011). To balance model fit and predictive ability, we conducted species-specific tuning of MAXENT model settings using AICc and the R package ENMeval (Muscarella et al., 2014) (for further details on MAXENT settings see Appendix S1).

We used MAXENT’s jackknife procedure to assess the relative importance of each variable (i.e., WBAL, *T*_min_ and GDD). Briefly, the jackknife tool excludes one variable at a time before running a model, thus testing model performance when different combinations of variables are included. MAXENT then compares the different model results and includes the variables that provide strong individual effects in the final model, and quantifies the relative importance of each variable in the final model.

Projecting models based on current climatic conditions may involve extrapolating model predictions beyond the observed data. To determine the extent to which our models were extrapolating, we conducted a multivariate environmental similarity surfaces (MESS) analysis (Elith, Kearney & Phillips, 2010). MESS analysis compares the reference climate (defined as the background regions) with the projected region by assigning negative values to sites where at least one input variable has a value outside of the reference climate (Elith et al., 2011; Webber et al., 2011).

We assessed model performance using Area Under the Curve (AUC) as calculated by MAXENT for the test data (Phillips, Anderson & Schapire, 2006; Ward, 2007). AUC values range from 0.5 (i.e., random) for models with no predictive ability to 1.0 (i.e., perfect discrimination between suitable and unsuitable cells) for models with a perfect prediction. According to the classification of Swets (1988), an AUC score of >0.7 (“useful” discrimination ability), >0.8 (“good” model performance) and >0.9 (“very good” model performance) was used. Since models were built for presence-only data, the background data constitute pseudo-absences for the AUC calculations (Phillips & Dudik, 2008).

To estimate the potential future allergy problems caused by ragweed expansion in Europe, we identified the areas, which under the models receive a future climate corresponding to the highest quartile (25%) of present day climate suitability for each of the three species. We refer to these as ‘high allergy risk’ (HAR) areas. The rationale for this approach was two-fold. First, the highest quartile of present-day climate suitability largely correspond to those areas in Europe where high levels of ragweed induced allergies are reported today (based on Déchamp, Méon & Reznik (2009) and Essl et al. (2015)). Second, we consider this approach to be a conservative one relative to predicting future potential allergy risk, which is preferable given the uncertainties that are always associated with forecasts based on modelling approaches. To estimate the potential future expansion of HAR areas, we reclassified raster maps into two classes defined by a break value corresponding to the lower limit of the highest quartile of present day habitat suitability. The reclassified raster maps were then used to calculate the change in HAR area between the present and the future under the RCP 6.0 and RCP 8.5 scenarios. We conducted these analyses in R (R Core Team, 2015) using the dismo (Hijmans et al., 2011) and raster (Hijmans & Van Etten, 2013) packages.

## Results

Based on all data points from the native range (Table 1), the median AUC values ranged from 0.80 to 0.82, depending on the species, indicating ‘good’ performance (AUC > 0.8) of all models for all three species (Table 2).

### Future species distribution in Europe

**Figure 1:**
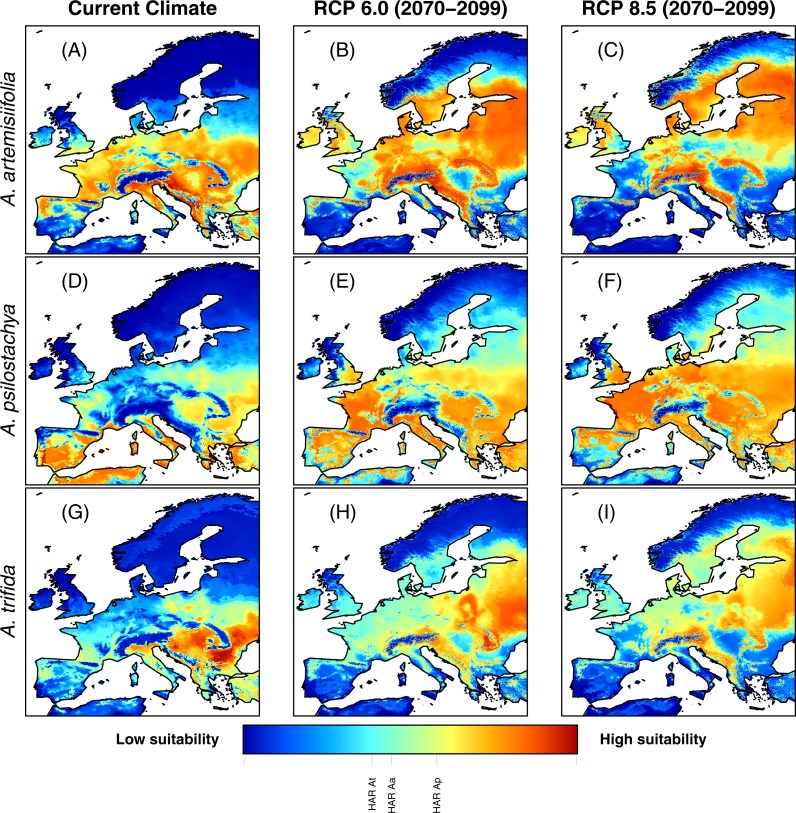
Habitat suitability of common ragweed (*A. artemisiifolia*) (A–C), perennial ragweed (*A. psilostachya*) (D–F) and giant ragweed (*A. trifida*) (G–I) in Europe under current climate conditions, and future climates (projections for years 2070–2099) assuming RCP 6.0 and RCP 8.5. Maps show average MAXENT values, derived from 15 replicates. Thresholds for HAR areas for each species are indicated graphically on the color bar (e.g., “HAR Aa” shows the HAR threshold value for *A. artemisiifolia*).

In Europe, all models predicted that climatically suitable areas for ragweed will become more widespread on the continent by year 2100 (following IPCC climate change scenarios RCP 6.0 and RCP 8.5) (Fig. 1). At present, the ragweed ‘high allergy risk’ (HAR) areas are found mostly in Southern Europe (Fig. 1 and Fig. S4). The models show a substantial increase of HAR areas across Europe by 2100 (Table 3). For common ragweed the HAR areas with maximum habitat suitability increased 100% (RCP 6.0) and 45% (RCP 8.5), respectively, with most new areas located in Northern (e.g., Denmark, United Kingdom) and Eastern Europe (e.g., the Baltic countries) (Fig. 1 and Table 3). For perennial ragweed models predicted an increase of 127% (RCP 6.0) and 189% (RCP 8.5), with new areas located across most of Central and Eastern Europe (Fig. 1 and Table 3). For giant ragweed, the HAR area increased by 97% for the RCP 6.0 projections but decreased by 69% for the RCP 8.5 projections. New habitats occurred especially in Russia, the Baltic countries and Southeastern Europe (Fig. 1 and Table 3).

**Table 3:** The area (km^2^) in future ‘high allergy risk’ (HAR) areas of common ragweed (*Ambrosia artemisiifolia*), perennial ragweed (*A. psilostachya*) and giant ragweed (*A. trifida*) in Europe under current and future climate (year 2100) conditions assuming RCP 6.0 and RCP 8.5 climate change scenarios (IPCC, 2014).

Species	Highest habitat suitability (km^2^)		
	Current	RCP 6.0	RCP 8.5
Common ragweed	89,460	178,960	129,320
Perennial ragweed	41,660	52,760	78,650
Giant ragweed	38,220	75,290	26,530

### Effects of environmental variables

**Figure 2:**
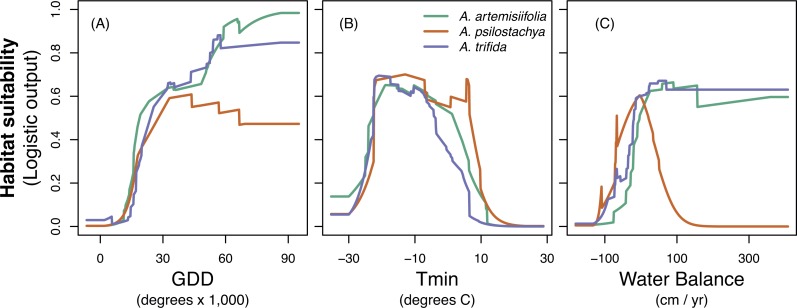
Response curves for common ragweed (*A. artemisiifolia*), perennial ragweed (*A. psilostachya*) and giant ragweed (*A. trifida*) to the three climatic variables; (A) growing degree days (GDD), (B) absolute minimum temperature (*T*_min_), and (C) water balance. Habitat suitability (given in MAXENT logistic output values) is averaged across 15 replicates.

The jackknife evaluation procedure suggested that GDD was the most important predictor for all three species. *T*_min_ was the second most important variable for perennial and giant ragweed, and WBAL was the second most important variable for common ragweed (Table 4 and Fig. S5). The response of the variables showed somewhat different tendencies (Fig. 2). Habitat suitability for all species tended to increase with GDD. Species showed similar response to *T*_min_. Habitat suitability of common ragweed and giant ragweed showed a similar increasing response to WBAL, whereas habitat suitability for perennial ragweed showed a hump-shaped relationship with WBAL values ranging from ca. −1,000 to 1,000 mm. Across species, WBAL had the strongest impact for common ragweed (Table 4). All species had their highest estimated habitat suitability for *T*_min_ values ranging from approximately −30 to 10 °C (Fig. 2). MESS comparison of the environmental factors in Europe indicated very little extrapolation outside the range of the native conditions. Specifically, more than 99% of grid cells for both the present-day and future climate conditions fell within the range of the native climate conditions (Fig. S6).

**Table 4:** Explanatory power of three climate variable (growing degree days, GDD; minimum temperature, *T*_min_; water balance, WBAL) deviated from the jackknife analysis on native range models of 15 replicates. Original jackknife analysis output of the model are found in Fig. S5.

Parameter	Species		
	Common ragweed	Perennial ragweed	Giant ragweed
GDD	0.425	0.456	0.548
*T*_min_	0.215	0.306	0.252
WBAL	0.36	0.238	0.2

## Discussion

Understanding the future distribution of allergenic plant species is crucial for predicting the consequences of the associated health related issues. Our study shows that three allergenic ragweed species have considerable potential to successfully expand their distribution range in Europe by the end of the century. The predicted ‘high allergy risk’ (HAR) areas, i.e., areas with climatic conditions corresponding to the highest quartile (25%) of present day habitat suitability for each of the three species, may expand extensively in Europe, depending on species and future climate change (RCP 6.0/8.5) scenarios (Table 3).

Our results for common ragweed agree with those of Cunze, Leiblein & Tackenberg (2013) in predicting an increased habitat suitability of common ragweed in Europe, particularly towards Northern and Eastern Europe. These authors, however, did not quantify the extent of the increase in areas where allergenic problems may be expected as a consequence of climatic changes. The majority of ragweed-studies so far have exclusively investigated common ragweed. However, considering that pollen of ragweed species is morphologically similar and give similar allergenic reactions (Frenz, 2001; Ziska et al., 2011; Sikoparija et al., 2016), it is important to consider multiple ragweed species and climate scenarios when predicting future increases in habitat suitability of allergenic ragweeds. Specifically, depending on the species and RCP used, our models estimated a change in ragweed HAR areas ranging from a loss of 11,690 km^2^ (giant ragweed, RCP 8.5) to an increase of 89,500 km^2^ (common ragweed RCP 6.0), affecting in particular Great Britain, Denmark, Sweden and the Baltic countries. Surprisingly, the more severe climate change scenario (RCP 8.5) did not facilitate an expansion of ragweeds further north compared to the milder scenario (RCP 6.0). In fact, the largest extent of suitable habitats was found under RCP 6.0 for both common and giant ragweed. We note that the northward expansion of ragweed species will be controlled by biological constraints in addition to climate variables (Deen, Hunt & Swanton, 1998). For instance, ragweeds are short-day plants and their flowering is induced by a dark period of approximately eight hours (Deen, Hunt & Swanton, 1998). Ragweeds are also sensitive to frost, and will not continue to flower when temperatures drop below freezing (Deen, Hunt & Swanton, 1998). In Northern Europe, growing season length and dark period duration may become insufficient for ragweed species to reproduce despite relatively favorable climatic conditions. Additionally, the lower extent of high habitat suitability for these species under RCP 8.5 could also be associated with drought. We found that both common and giant ragweed perform well under relatively wet conditions, whereas perennial ragweed performs better in drier habitats (Fig. 2). Prolonged drought and dry periods are increasingly expected under RCP 8.5, hence water scarcity provides one potential explanation why perennial ragweed will become most abundant under RCP 8.5 compared to both common and giant ragweed. Overall, we recommend that further work examining ragweed species distributions may be improved by considering important non-climatic variables including, for example, day length.

In addition to a geographical increase in areas with high climate suitability, a warmer climate may facilitate flowering and pollen production in areas where flowering and seed maturation currently is limited by low temperatures. Thus, allergenic pollen load could also be intensified within the current realized range of the three ragweed species. These changes impose substantial health related risks, as evident from current HAR areas in Southern Europe. For example, a prick-test study of ragweed allergens showed levels of pollen-allergic patients ranging from 30–40% in the Rhône Valley (France), to 70% in Northern Italy and more than 80% in Hungary (Rybnícek & Jäger, 2001). Another health related concern is cross-reactivity between species within the genus *Ambrosia* as well as with other allergenic species, such as mugwort (*Artemisia* L.) species (White & Bernstein, 2003; D’Amato et al., 2007). This combined with the late flowering of ragweeds, that will prolong the local pollen season (D’Amato et al., 2007; Vogl et al., 2008), are likely to entail medical and socio-economical implications for the public in novel HAR areas.

In the context of allergenic plants, urban areas are of main concern due to their high population densities (higher exposure risk) and elevated CO_2_ concentrations that are known to facilitate biomass and pollen production (Ziska et al., 2003). Our models indicate that future ragweed HAR areas will overlap with major cities in Europe including St. Petersburg, Hamburg and Paris. Ragweeds future distribution may therefore affect large populations across Europe. A higher overall abundance of ragweed species also increases the risk of long-distance transport of allergenic pollen (Grewling et al., 2016). Ragweed pollen can disperse 100’s of kilometers (Bullock et al., 2013), thus affecting allergic persons in areas without established ragweed populations. This has already been shown using complex cluster backward trajectories (Makra et al., 2016), and Hamaoui-Laguel et al. (2015) estimated that airborne ragweed pollen concentration will increase about four times by 2050, dispersing pollen across large regions of Europe. The low atmospheric concentration of ragweed pollen necessary to induce allergic reactions in sensitive patients (∼5 grains m^−3^) (Déchamp et al., 1997) combined with the long-distance wind dispersal potential, makes urban ragweed populations important sources for pollen induced allergies across large spatial areas (Belmonte et al., 2000; Essl et al., 2015; Hamaoui-Laguel et al., 2015).

While the results of this study can inform future management strategies addressing climate change and increasing human disturbance, there is a need to acknowledge the caveats embedded in all climate model studies (Araujo & Guisan, 2006). For instance, our model does not take into consideration anthropogenic factors like disturbance, land use and propagule pressure, which will be crucial in controlling range filling within the potential range set by the climatic variables. Including such parameters in future model-work will allow us to expand knowledge and predictability of the distribution of invasive allergenic species (Chapman et al., 2016). Local adaptation is another parameter that may affect the distribution of species and their response to climate change. Intra-population local adaption has been shown to allow species to survive in otherwise unfavorable conditions (Li et al., 2015). Thus, incorporation of physiological performance/phenology of ragweed throughout their distribution would be beneficial in future work to estimate range expansion at regional or local scales (Chapman et al., 2014). For now, our results demonstrate that future climate changes could result in a large expansion of *Ambrosia* related allergy problems in Europe, unless precautionary efforts are made to limit further expansion of the three species across Europe (Pearson, 2007; Webber et al., 2011; Chapman et al., 2016). In Northern and Eastern Europe, focus should primarily be on restricting further invasion and establishment of ragweed (Dullinger et al., 2009; Cunze, Leiblein & Tackenberg, 2013; Storkey et al., 2014). The most important factor for successful invasion by ragweed is propagule pressure, both in terms of number of introductions and number of propagules released (Lockwood, Cassey & Blackburn, 2005; Gladieux et al., 2011). Ragweeds are species that propagate exclusively by seeds. Long-distance dispersal of ragweed seeds is mostly dependent on anthropogenic forces, including import of contaminated birdseeds and crops (e.g., maize and soya beans), transport of contaminated soil, and seed infested agricultural machines (Essl et al., 2015; Chapman et al., 2016). Seeds may remain dormant for as long as 40 years (Bassett & Crompton, 1975) if conditions are unfavorable, calling for a long term effort in controlling ragweed invasion. All these aspects illustrate the importance of cross-countries management planning in order to prevent and control future spread of these allergenic bioinvaders.

##  Supplemental Information

10.7717/peerj.3104/supp-1 Figure S1 Distribution records of three ragweeds in Europe Maps showing occurrence records of *A. artemisiifolia, A. psilostachya* and *A. trifida*. Points represent the ‘cleaned’ species occurrence records (*see main text*). The points within the outlined frame illustrate the native dataset, whereas all points illustrate the global dataset.

10.7717/peerj.3104/supp-2 Figure S2 Models trained on distribution records in Europe Habitat suitability of common ragweed (*A. artemisiifolia*) (A–C)*,* perennial ragweed** (*A. psilostachya*) (D–F) and giant ragweed (*A. trifida*) (G–I) in Europe under current climate conditions, and future climates (projections for years 2070–2099) assuming RCP 6.0 and RCP 8.5. Maps show average MAXENT values, derived from 15 replicates.

10.7717/peerj.3104/supp-3 Figure S3 Models trained on distribution records in Europe and North America combined Habitat suitability of common ragweed (*A. artemisiifolia*) (A–C)*,* perennial ragweed (*A. psilostachya*) (D-F) and giant ragweed (*A. trifida*) (G-I) in Europe under current climate conditions, and future climates (projections for years 2070-2099) assuming RCP 6.0 and RCP 8.5. Maps show average MAXENT values, derived from 15 replicates.

10.7717/peerj.3104/supp-4 Figure S4 High Risk Allergy areas in Europe High allergy risk’ (HAR) areas of common ragweed *A. artemisiifolia*) (A–C), perennial ragweed (*A. psilostachya*) (D–F) and giant ragweed (*A. trifida*) (G–I) in Europe under current climate conditions, and projected future climates (for years 2070–2099) under RCP 6.0 and RCP 8.5. Letters indicate locations of major cities (a, Madrid; b, London; c, Paris; d, Hamburg; e, Rome; f, Berlin; g, Vienna; h, Bucharest; i, Istanbul; j, Saint Petersburg).

10.7717/peerj.3104/supp-5 Figure S5 Jackknife results Original jackknife model output.

10.7717/peerj.3104/supp-6 Figure S6 MESS analysis results Figure displaying the MESS analysis results.

10.7717/peerj.3104/supp-7 Data S1 Climatic data for *A. psilostachya*

10.7717/peerj.3104/supp-8 Data S2 Climatic data for *A. trifida*

10.7717/peerj.3104/supp-9 Data S3 Climatic data for *A. artemisiifolia*

10.7717/peerj.3104/supp-10 Supplemental Information 1 Native range occurrence for *A. trifida*

10.7717/peerj.3104/supp-11 Supplemental Information 2 Native range occurrence for *A. artemisiifolia*

10.7717/peerj.3104/supp-12 Supplemental Information 3 Native range occurrence for a psilostachya

10.7717/peerj.3104/supp-13 Supplemental Information 4 Text for supplementary tables and files Supplementary material presenting additional information on (1) the method and (2) extended results not included in the paper.

## Additional Information and Declarations

### Data Availability

The following information was supplied regarding data availability: The raw data has been supplied as a Supplemental Information.
