# Supplementary figures and images for: Climate-change-induced range shifts of three allergenic ragweeds (Ambrosia L.) in Europe and their potential impact on human health
Source: PeerJ. 2017 Mar 16;5:e3104. doi: 10.7717/peerj.3104 (PMC5357339; doi:10.7717/peerj.3104)

*A. artemisiifolia*

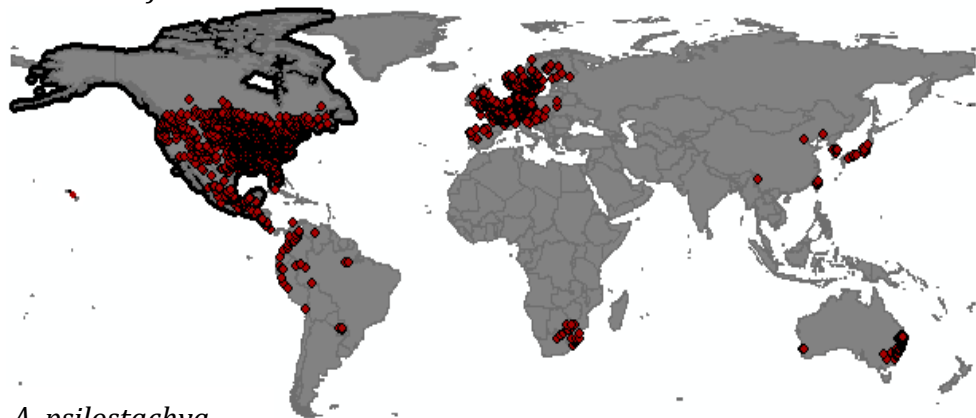

*A. psilostachya*

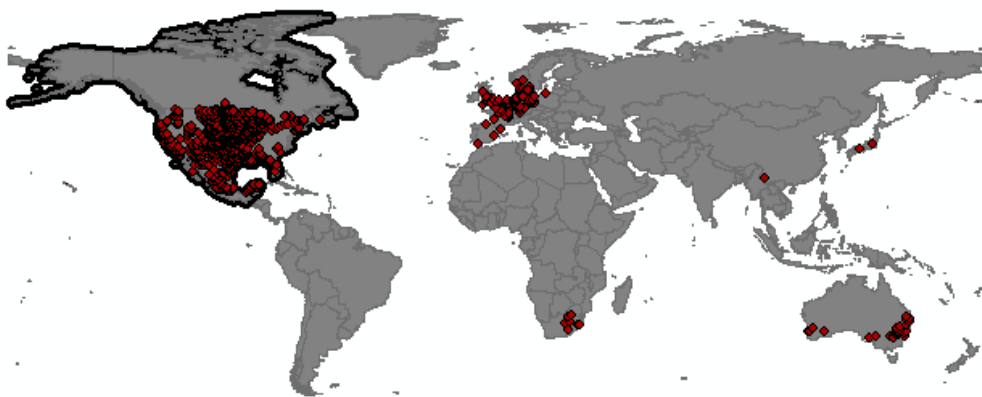

*A. trifida*

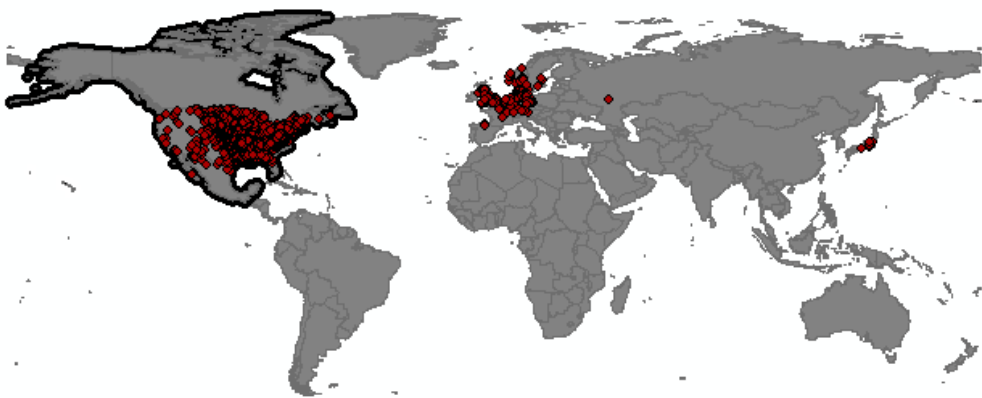

Supplement: Figure S1 — Maps showing occurrence records of A. artemisiifolia, A. psilostachya and A. trifida. Points represent the ‘cleaned’ species occurrence records (see main text). The points within the outlined frame illustrate the native dataset, whereas all points illustrate the global dataset. [file peerj-05-3104-s001.pdf]

# Current Climate

# RCP 6.0 (2070–2099)

# RCP 8.5 (2070–2099)

*A. artemisiifolia*

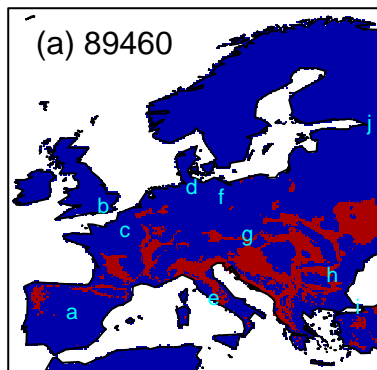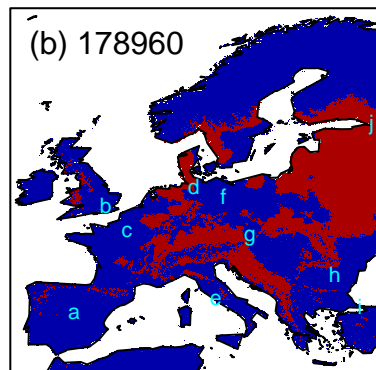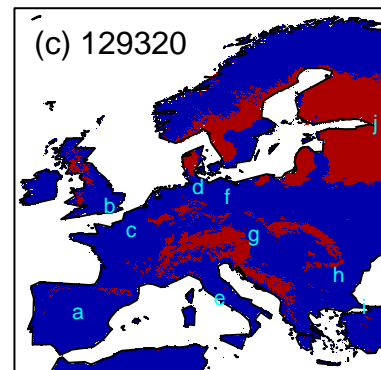

*A. psilostachya*

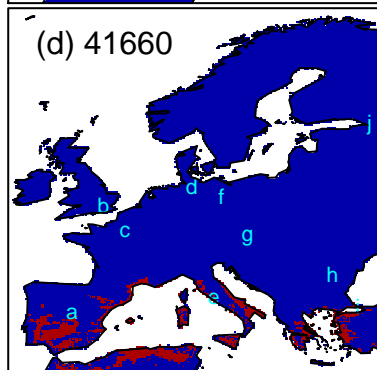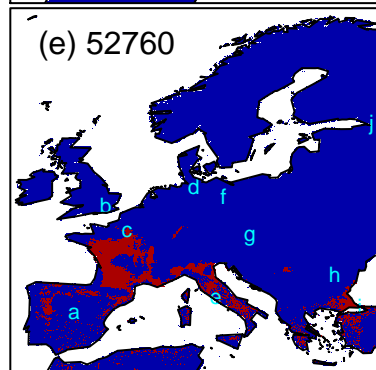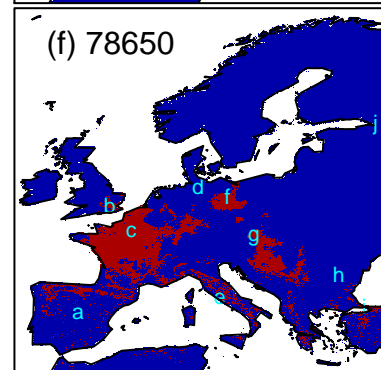

*A. trifida*

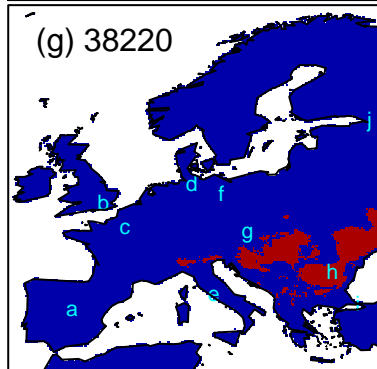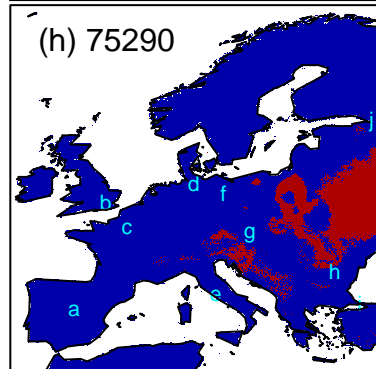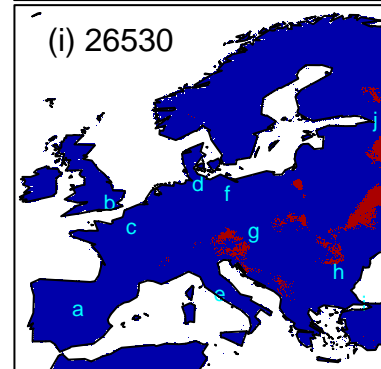

Non-HAR Area

HAR area

Supplement: Figure S4 — High allergy risk’ (HAR) areas of common ragweed A. artemisiifolia) (A–C), perennial ragweed (A. psilostachya) (D–F) and giant ragweed (A. trifida) (G–I) in Europe under current climate conditions, and projected future climates (for years 2070–2099) under RCP 6.0 and RCP 8.5. Letters indicate locations of major cities (a, Madrid; b, London; c, Paris; d, Hamburg; e, Rome; f, Berlin; g, Vienna; h, Bucharest; i, Istanbul; j, Saint Petersburg). [file peerj-05-3104-s004.pdf]

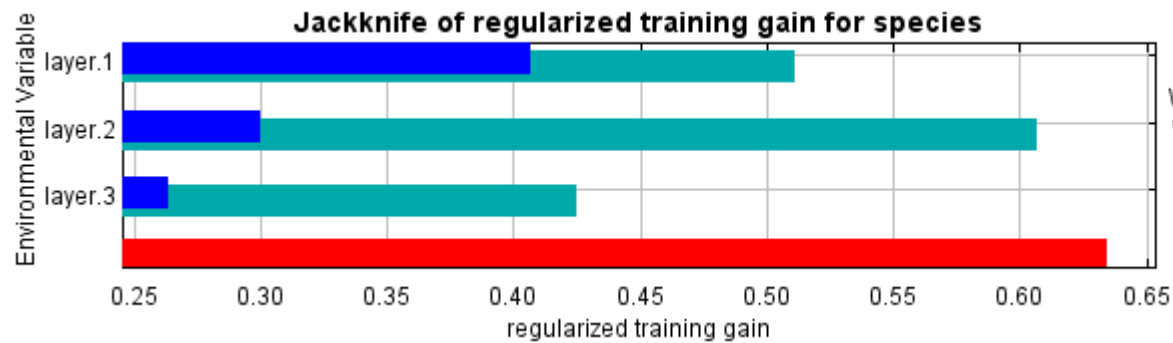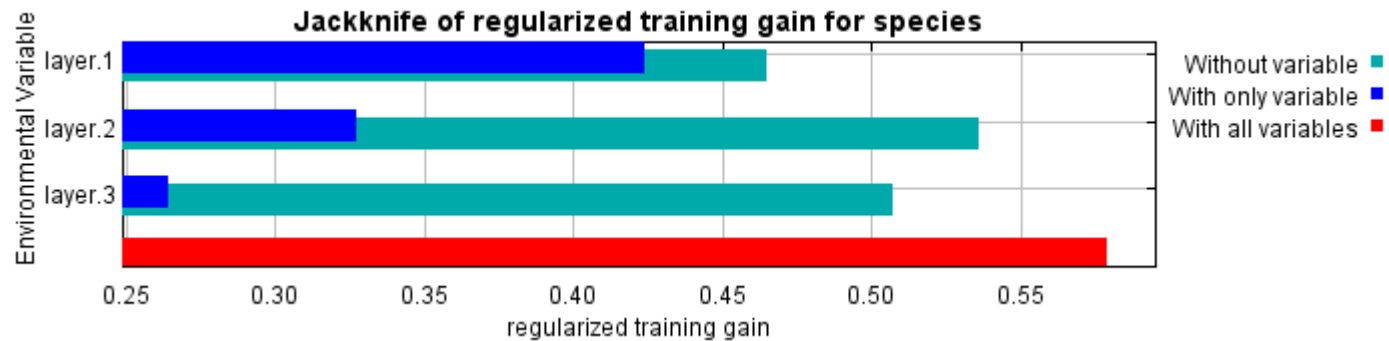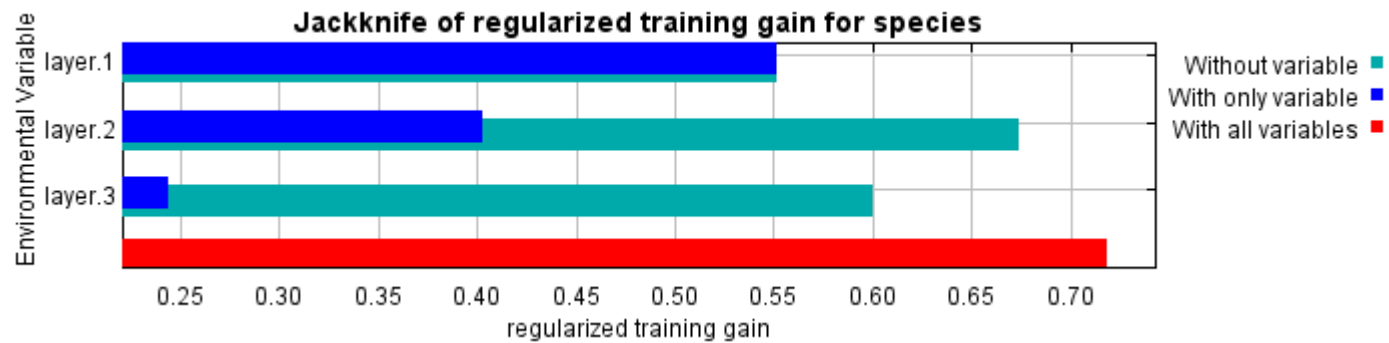

Supplement: Figure S5 — Original jackknife model output. [file peerj-05-3104-s005.pdf]
