# Supplementary material for: Climate-change-induced range shifts of three allergenic ragweeds (Ambrosia L.) in Europe and their potential impact on human health
Source: PeerJ. 2017 Mar 16;5:e3104. doi: 10.7717/peerj.3104 (PMC5357339; doi:10.7717/peerj.3104)
Supplement: Figure S2 — Habitat suitability of common ragweed (A. artemisiifolia) (A–C), perennial ragweed (A. psilostachya) (D–F) and giant ragweed (A. trifida) (G–I) in Europe under current climate conditions, and future climates (projections for years 2070–2099) assuming RCP 6.0 and RCP 8.5. Maps show average MAXENT values, derived from 15 replicates. [file peerj-05-3104-s002.pdf]

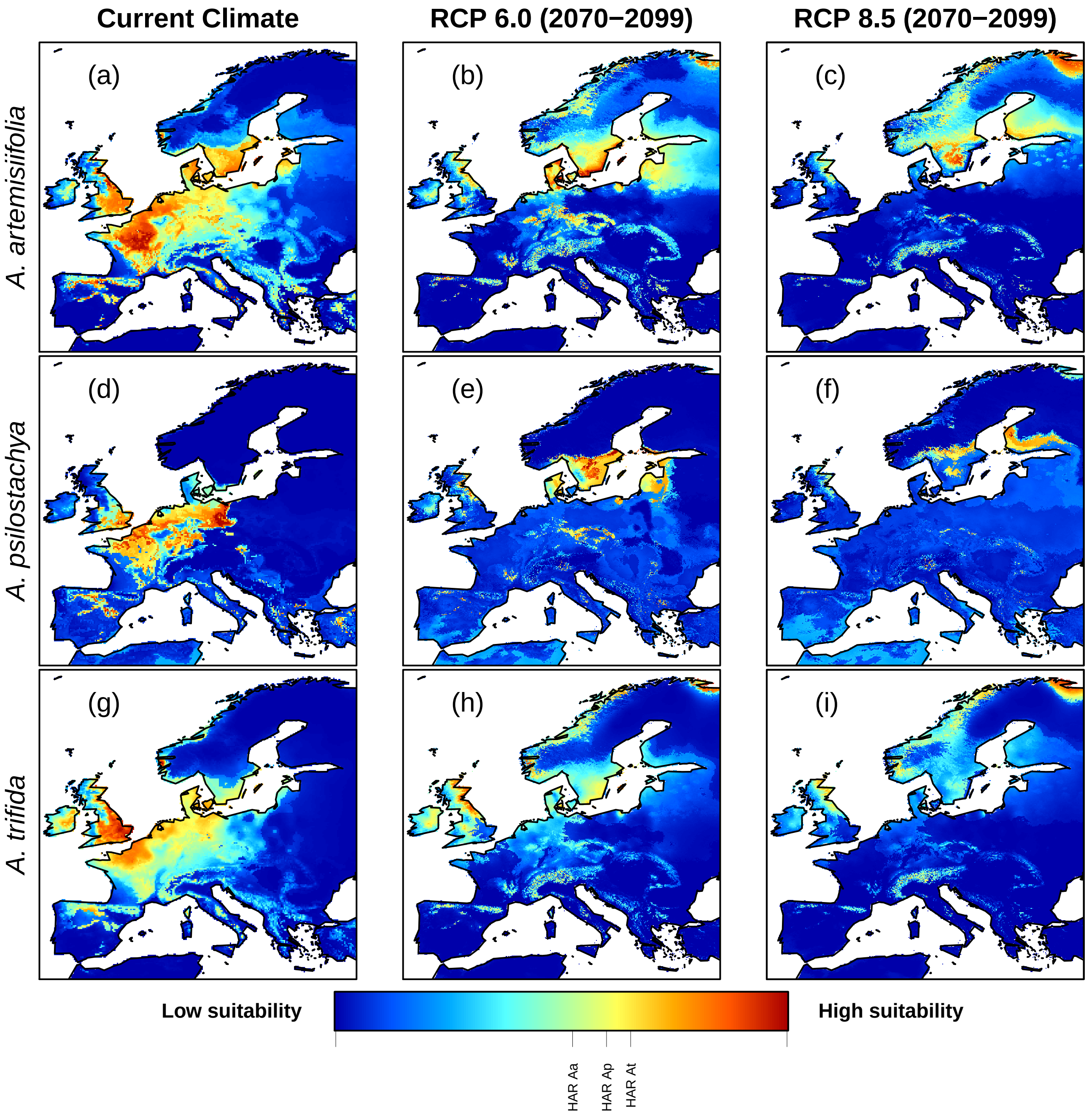

Current Climate
RCP 6.0 (2070−2099)
RCP 8.5 (2070−2099)
A. artemisiifolia
A. psilostachya
A. trifida
(a)
(b)
(c)
(d)
(e)
(f)
(g)
(h)
(i)
Low suitability
High suitability
HAR Aa
HAR Ap
HAR At
