# Supplementary material for: Climate-change-induced range shifts of three allergenic ragweeds (Ambrosia L.) in Europe and their potential impact on human health
Source: PeerJ. 2017 Mar 16;5:e3104. doi: 10.7717/peerj.3104 (PMC5357339; doi:10.7717/peerj.3104)
Supplement: Figure S3 — Habitat suitability of common ragweed (A. artemisiifolia) (A–C), perennial ragweed (A. psilostachya) (D-F) and giant ragweed (A. trifida) (G-I) in Europe under current climate conditions, and future climates (projections for years 2070-2099) assuming RCP 6.0 and RCP 8.5. Maps show average MAXENT values, derived from 15 replicates. [file peerj-05-3104-s003.pdf]

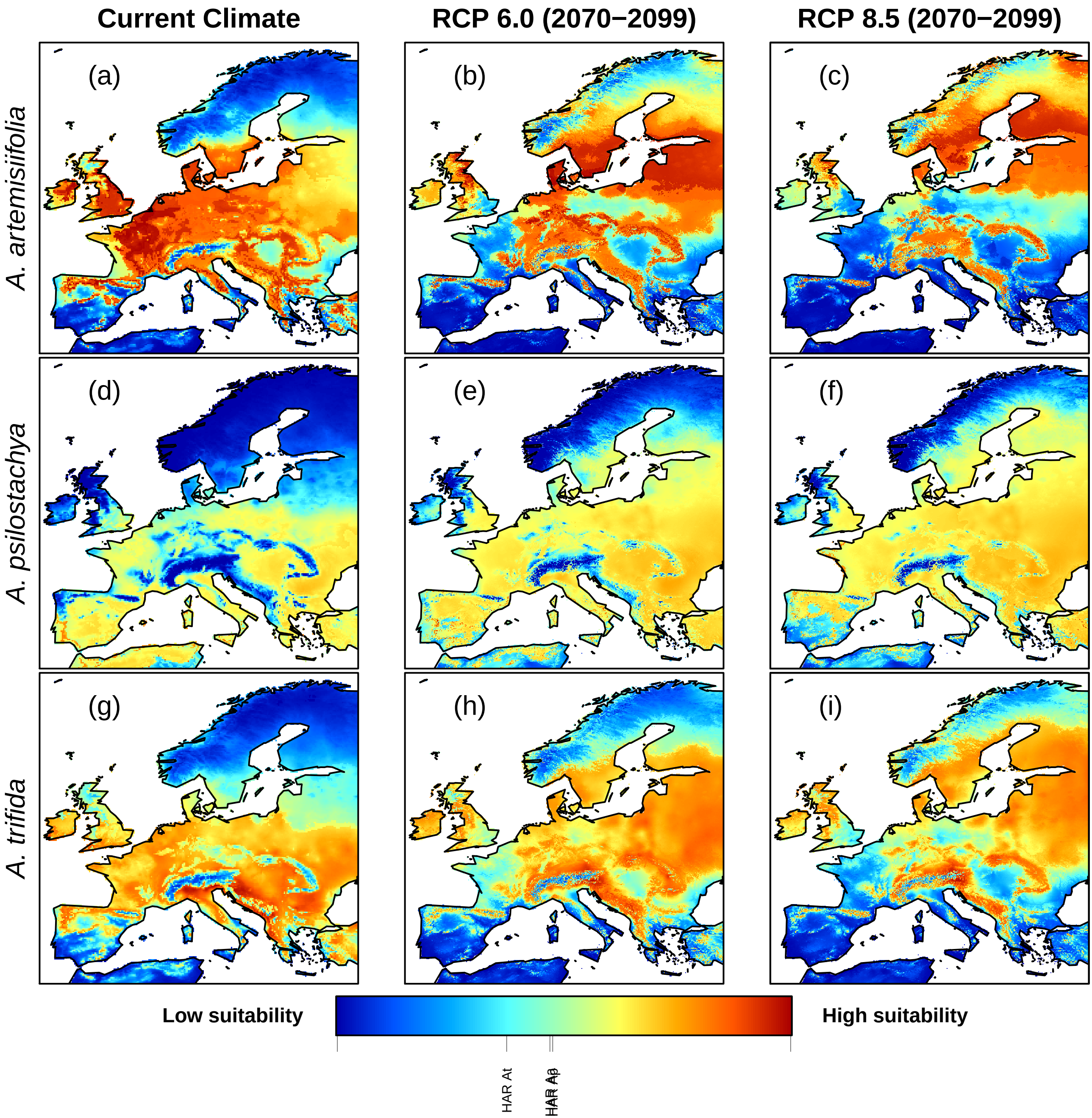

Current Climate
RCP 6.0 (2070−2099)
RCP 8.5 (2070−2099)
A. artemisiifolia
A. psilostachya
A. trifida
(a)
(b)
(c)
(d)
(e)
(f)
(g)
(h)
(i)
Low suitability
High suitability
HAR At
HAR Ap
