# Supplementary material for: Climate-change-induced range shifts of three allergenic ragweeds (Ambrosia L.) in Europe and their potential impact on human health
Source: PeerJ. 2017 Mar 16;5:e3104. doi: 10.7717/peerj.3104 (PMC5357339; doi:10.7717/peerj.3104)
Supplement: Figure S6 — Figure displaying the MESS analysis results. [file peerj-05-3104-s006.pdf]

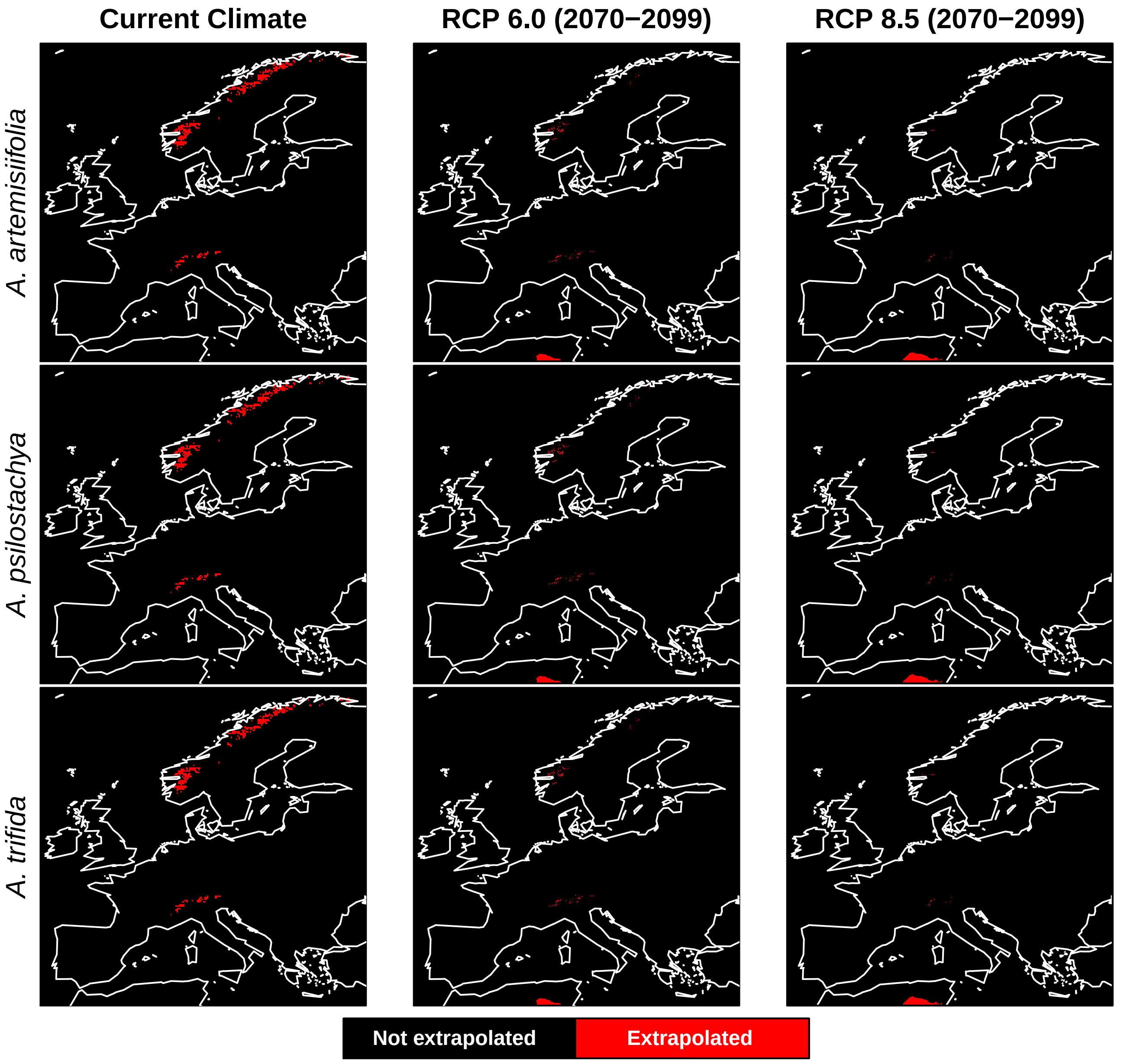
Current Climate
RCP 6.0 (2070−2099)
RCP 8.5 (2070−2099)
A. artemisiifolia
A. psilostachya
A. trifida
Not extrapolated
Extrapolated
