# Supplementary material for: Climate-change-induced range shifts of three allergenic ragweeds (Ambrosia L.) in Europe and their potential impact on human health
Source: PeerJ. 2017 Mar 16;5:e3104. doi: 10.7717/peerj.3104 (PMC5357339; doi:10.7717/peerj.3104)
Supplement: Supplemental Information 4 — Supplementary material presenting additional information on (1) the method and (2) extended results not included in the paper. [file peerj-05-3104-s013.docx]

**Supplementary material**

**Climate change induced range shifts of three allergenic ragweeds (*Ambrosia* L.) in Europe and potential impact on human health**

Karen Rasmussen^1,†,§,*^, Jakob Thyrring^2,§^, Robert Muscarella^1^, Finn Borchsenius^3^

^1^ Section for Ecoinformatics & Biodiversity, Department of Bioscience, Aarhus University, Ny Munkegade 116, building 1540, DK-8000, Aarhus C, Denmark

^2^ Arctic Research Centre, Department of Bioscience, Aarhus University, Ny Munkegade 114, building 1540, DK-8000, Aarhus C, Denmark

^3^ Science Museums, Aarhus University, Ole Worms Allé 1, building 1137, DK-8000, Aarhus C, Denmark

^†^*Present address:* Asthma-Allergy Denmark, Universitetsparken 4, DK-4000, Roskilde, Denmark

^*^ Corresponding author: kr@astma-allergi.dk

^§^ Shared lead authorship

**Figure S1** Maps showing occurrence records of *A. artemisiifolia, A. psilostachya* and *A. trifida*. Points represent the ‘cleaned’ species occurrence records (*see main text*). The points within the outlined frame illustrate the native dataset, whereas all points illustrate the global dataset.

*
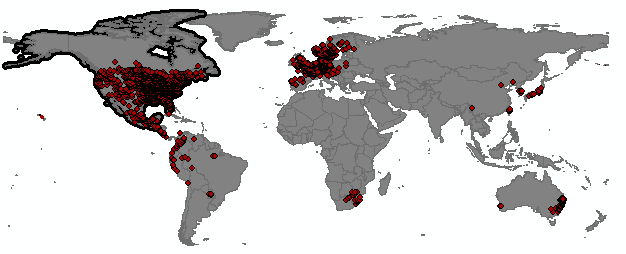
*

*A. trifida*


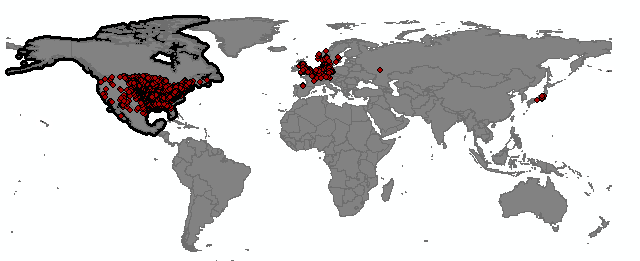

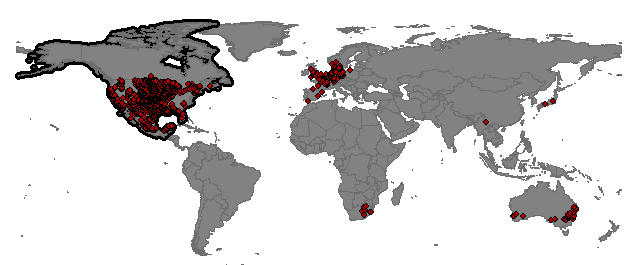


*A. psilostachya*

*A. artemisiifolia*

**Table S1** Distribution of the ragweed species (outside their North American native range). Data sources: Global Invasive Species Database (www.iucngisd.org) for *A. artemisiifolia*, European and Mediterranean Plant Protection Organization (www.eppo.int) and Delivering Alien Invasive Species Inventories for Europe (www.europe-aliens.org) for *A. artemisiifolia, A. psilostachya* and *A. trifida*

| Species | Europe |  | Asia | Africa | Central, North- and South America |  | | Oceanaria | |
| --- | --- | --- | --- | --- | --- | --- | --- | --- | --- |
|  | Austria, Belgium, Bulgaria, Croatia, Czech Republic, Denmark, Estonia, Finland, France, Germany, Hungary, Italy, Latvia, Liechtenstein, Lithuania, Luxemburg, Moldova, Netherlands, Norway, Poland, Portugal, Romania, Russia (Krasnodar territory), Scotland, Slovakia, Slovenia, Spain, Sweden, Switzerland, Ukraine, United Kingdom, (former Yugoslavia)  Austria, Belgium, Croatia, Czech Republic, Denmark, England, Estonia, Finland, France, Germany, Hungary, Italy, Latvia, Netherlands, Norway, Poland, Russia (South of European Russia), Scotland, Spain, Sweden, Switzerland, United Kingdom    Austria, Belgium, Czech Republic, Denmark, France, Germany, Ireland, Italy, Latvia, Lithuania, Netherlands, Norway, Russia (South of European Russia), Scotland, Sweden, Switzerland, Ukraine, United Kingdom (Yugoslavia) | | Azerbaijan, China, India, Japan, Kazakhstan, Korea, Russia (Primorski territory), Taiwan, Turkey  Kazakhstan  Georgia, Japan,  Israel | Mauritius  Mauritius | Argentina, Brazil, Bolivia, Chile, Colombia, Cuba, Guadeloupe, Guatemala, Jamaica, Martinque, Paraguay, Peru, Uruguay | | Australia  New Zealand  Australia | |  |
| *A. artemisiifolia* |  |  |  |  |  |  |  |  |  |
| *A. psilostachya* |  |  |  |  |  |  |  |  |  |
|  |  |  |  |  |  |  |  |  |  |
| *A. trifida* |  |  |  |  |  |  |  |  |  |

**Table S2** Model predictive ability based on median Area Under the receiver operating Curve (AUC) values of the model in native range for common ragweed (*Ambrosia artemisiifolia*), perennial ragweed (*A. psilostachya*) and giant ragweed (*A. trifida*). AUC values were derived from average test AUC values for MAXENT models of 15 replicates based on occurrence records from the native North American range combined with records from the invasive European range and records from the invasive European range only.

|  | **Model AUC** | | |
| --- | --- | --- | --- |
|  | Common ragweed Perennial ragweed Giant ragweed | | |
| North American + European range | 0.74 0.77 0.79 | | |
| European range only | 0.81 | 0.91 0.87 |  |

**Appendix S1: Extended methods**

**Explanatory variables: {Riahi, 2007 #2428}**Three bioclimatic parameters, of known importance for the geographical distribution of plants, were used to describe the species climatic requirements in this study. Specifically, we used monthly values of mean temperature and precipitation from the CRU CL 2.0 dataset at a 10’ resolution (http://www.cru.uea.ac.uk/cru/data/hrg/; period 1961-1990; New et al. 2002) to derive the following variables: Growing Degree Days (GDD; computed with a 5°C base following Prentice et al. 1992, Zimmermann & Kienast 1999), Water Balance (WBAL; computed as the yearly sum of the monthly differences between precipitation and potential evapotranspiration, following Lugo et al. 1999, Skov & Svenning 2004) and Absolute Minimum Temperature (T_min_; estimated from the mean temperature of the coldest month after Prentice et al. 1992). Climate change projections used in this study were based on averages taken across all available global circulation models provided by the IPCC AR5 (IPCC) for representative concentration pathways (RCPs) 6.0 and 8.5 (Fujino et al. 2006; Riahi et al. 2007; Hijioka et al. 2008).

**MAXENT** **settings:**

We used ENMeval (Muscarella et al. 2014) for species-specific tuning of MAXENT models. Specifically, for each species, we built models with all combinations of regularization multiplier values ranging from 0.5 to 4 (in increments of 0.5) and all possible feature class combinations. For each model, we used the ‘checkerboard2’ method to partition data into test and training bins for evaluation. We then selected the ‘optimal’ model settings (regularization and feature classes) for each species based on the model with the lowest AICc. Then, we reran the model with the ‘optimal’ settings using 15 replicate runs. We used the following other settings: Jack-knife test = true, replicates = 15 (replicated run type = subsample), random seed = true, remove duplicate presence records = true, write plot data = true, extrapolate = false, maximum iterations = 5000.

**Figure S2 Models trained on distribution records in Europe.** Habitat suitability of common ragweed (*A. artemisiifolia*) (a-c)*,* perennial ragweed (*A. psilostachya*) (d-f) and giant ragweed (*A. trifida*) (g-i) in Europe under current climate conditions, and future climates (projections for years 2070-2099) assuming RCP 6.0 and RCP 8.5. Maps show average MAXENT values, derived from 15 replicates.


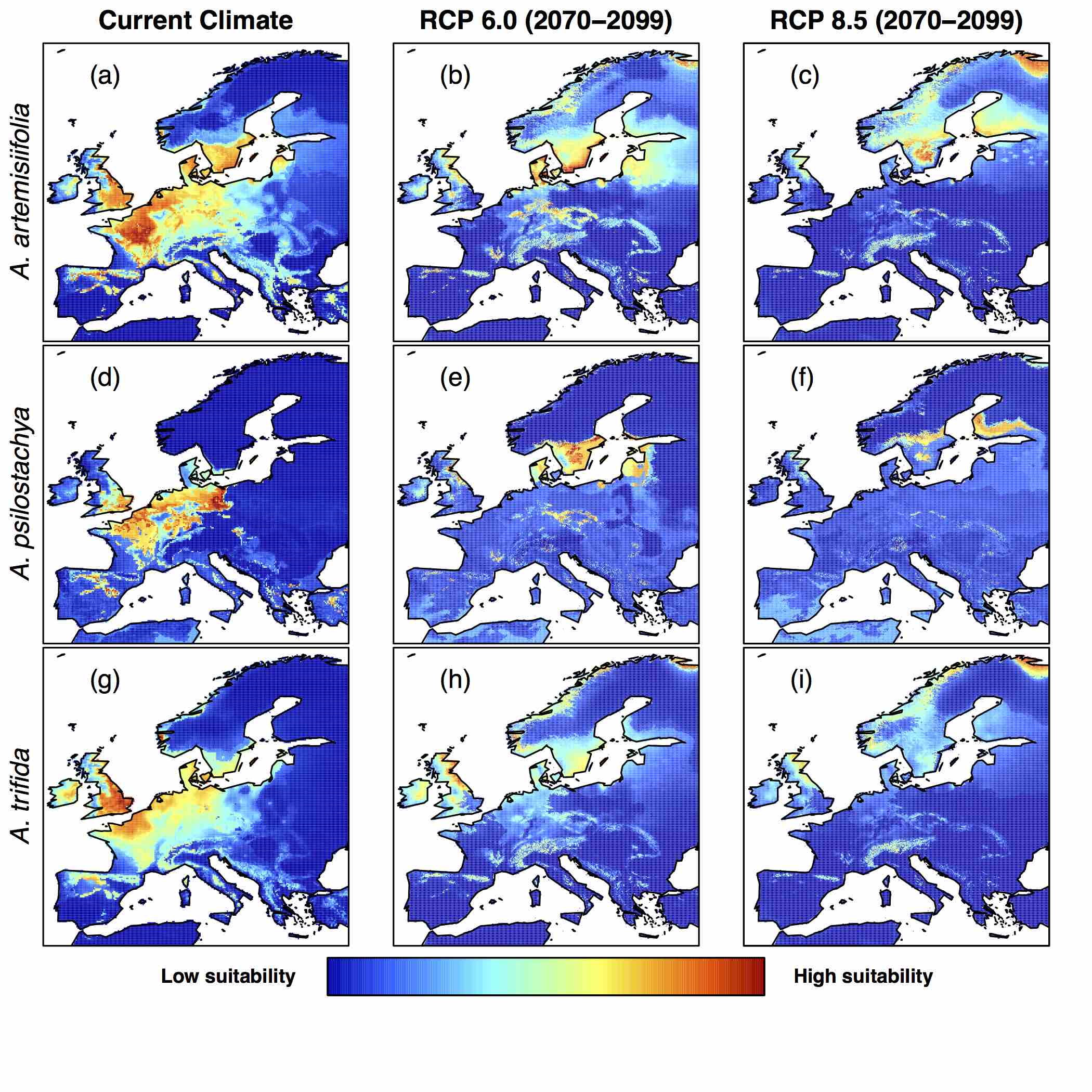


**Figure S3 Models trained on distribution records in Europe and North America combined.** Habitat suitability of common ragweed (*A. artemisiifolia*) (a-c)*,* perennial ragweed (*A. psilostachya*) (d-f) and giant ragweed (*A. trifida*) (g-i) in Europe under current climate conditions, and future climates (projections for years 2070-2099) assuming RCP 6.0 and RCP 8.5. Maps show average MAXENT values, derived from 15 replicates.


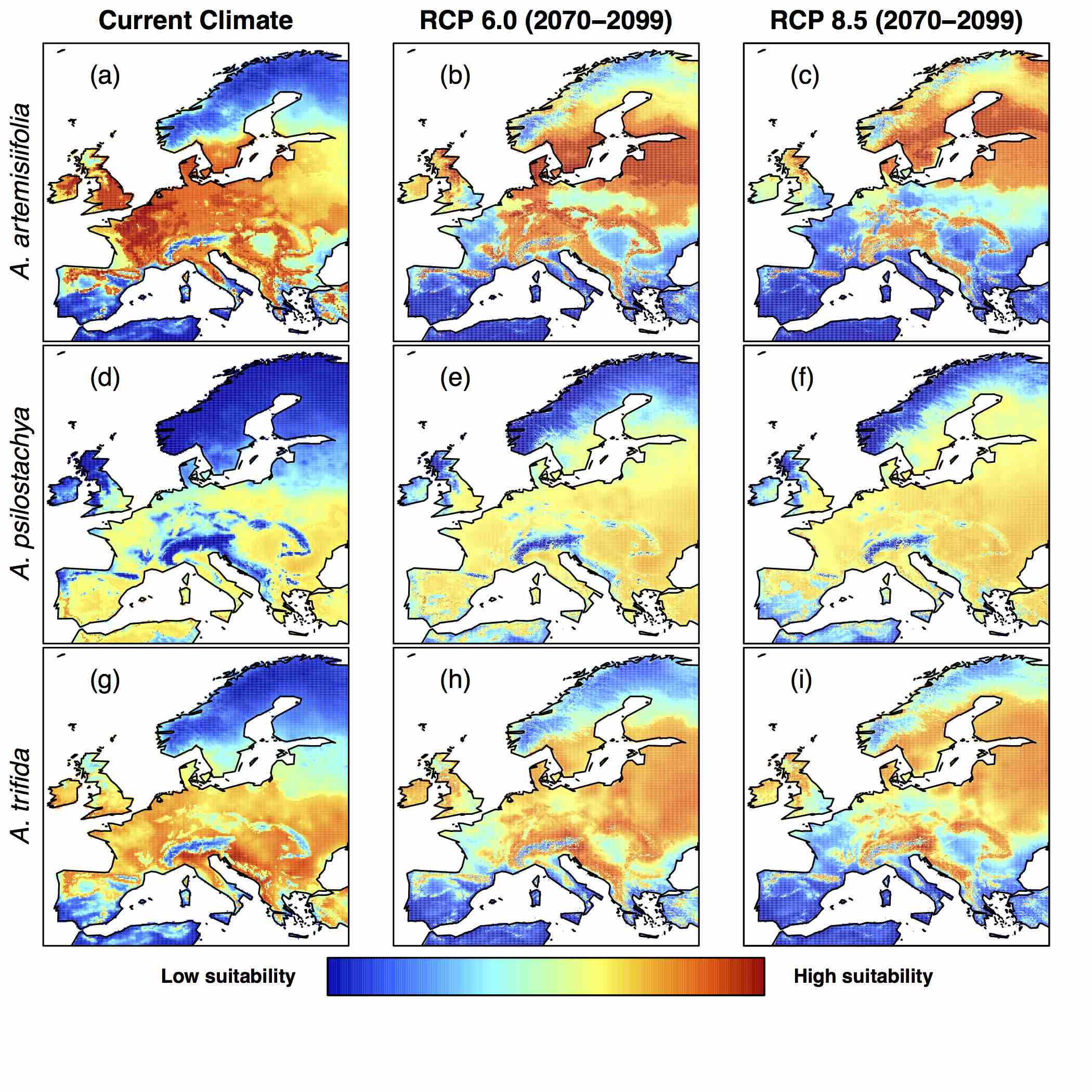


**Figure S4** High allergy risk’ (HAR) areas of common ragweed *A. artemisiifolia*) (a-c)*,* perennial ragweed (*A. psilostachya*) (d-f) and giant ragweed (*A. trifida*) (g-i) in Europe under current climate conditions, and projected future climates (for years 2070-2099) under RCP 6.0 and RCP 8.5. Letters indicate locations of major cities (a=Madrid, b=London, c=Paris, d=Hamburg, e=Rome, f=Berlin, g=Vienna , h=Bucharest, i=Istanbul, j=Saint Petersburg).

**
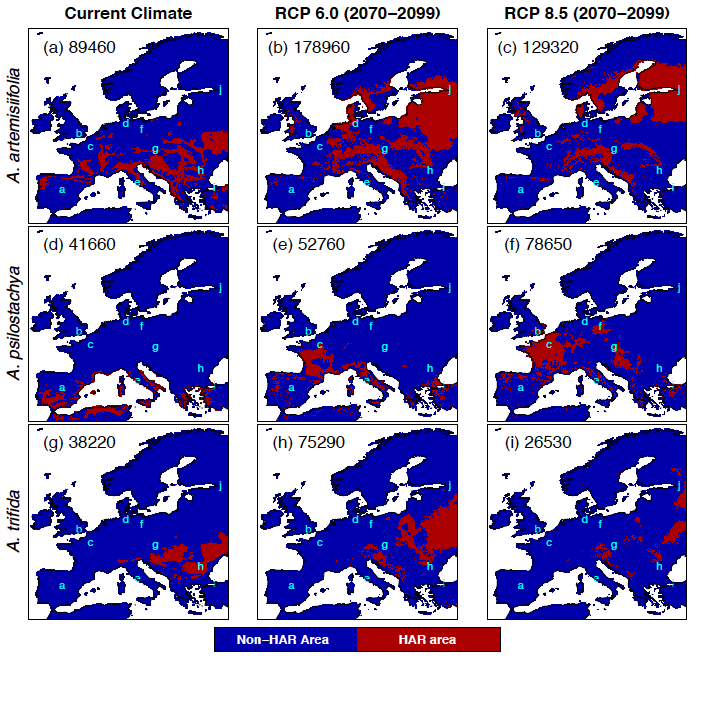
**

**Figure S5** Original jackknife results.

**
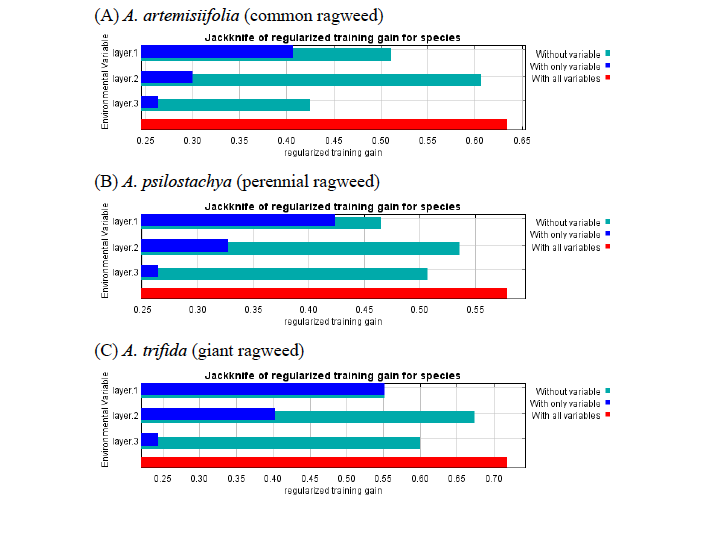
**

**Figure S6** Mess analysis results.


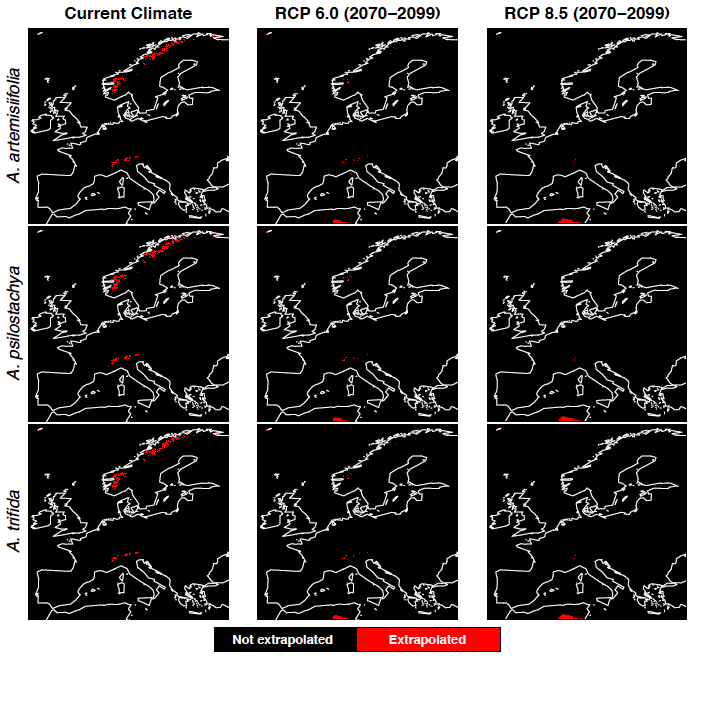


**Reference**

Fujino J, Nair R, Kainuma M, Masui T, and Matsuoka Y. 2006. Multi-gas Mitigation Analysis on Stabilization Scenarios Using Aim Global Model. *The Energy Journal* 27:343-353.

Hijioka Y, Y. M, H. N, M. M, and M. K. 2008. Global GHG emissions scenarios under GHG concentration stabilization targets. *Journal of Global Environmental Engineerin* 13:97-108.

Lugo, A.E., Brown, S.L., Dodson, R., Smith, T.S. & Shugart, H.H. (1999) The Holdridge life zones of the conterminous United States in relation to ecosystem mapping. *Journal of Biogeography*, **26**, 1025-1038.

New, M., Lister, D., Hulme, M. & Makin, I. (2002) A high-resolution data set of surface climate over global land areas. *Climate Research*, **21**, 1-25.

Muscarella R, Galante PJ, Soley-Guardia M, Boria RA, Kass JM, Uriarte M, and Anderson RP. 2014. ENMeval: An R package for conducting spatially independent evaluations and estimating optimal model complexity for Maxent ecological niche models. *Methods in Ecology and Evolution* 5:1198-1205.

Normand, S., Svenning, J.C. & Skov, F. (2007) National and European perspectives on climate change sensitivity of the habitats directive characteristic plant species. *Journal for Nature Conservation*, **15**, 41-53.

Prentice, I.C., Cramer, W., Harrison, S.P., Leemans, R., Monserud, R.A. & Solomon, A.M. (1992) A global biome model based on plant physiology and sominance, soil properties and climate. *Journal of Biogeography*, **19**, 117-134.

Riahi K, Grübler A, and Nakicenovic N. 2007. Scenarios of long-term socio-economic and environmental development under climate stabilization. *Technological Forecasting and Social Change* 74:887-935.

Skov, F. & Svenning, J.C. (2004) Potential impact of climatic change on the distribution of forest herbs in Europe. *Ecography*, **27**, 366-380.

Zimmermann, N.E. & Kienast, F. (1999) Predictive mapping of alpine grasslands in Switzerland: Species versus community approach. *Journal of Vegetation Science*, **10**, 469-4
